# Pneumocystis Pneumonia in a Non-AIDS Patient

**DOI:** 10.7759/cureus.83746

**Published:** 2025-05-08

**Authors:** Adel N Shalabi, Taha Saleem, Neel H Patel, Lauren E Blewett, James L Crowe

**Affiliations:** 1 Internal Medicine, Saba University School of Medicine, Devens, USA; 2 Internal Medicine, Leonard J. Chabert Medical Center, Houma, USA; 3 Infectious Diseases, Leonard J. Chabert Medical Center, Houma, USA

**Keywords:** hiv cd4 t-cells, hiv diseases, hiv infections, human immuno-deficiency virus (hiv), non-aids pcp, opportunist infections in hiv, pjp in hiv, pneumocystis carinii pneumonia, pneumocystis jiroveci pneumonia, pneumocystis pneumonia (pcp)

## Abstract

We present a unique case of *Pneumocystis jirovecii* pneumonia (PCP) in a patient with HIV. Both treated and untreated HIV patients are susceptible to opportunistic pathogens. Depending on the patient’s underlying cluster of differentiation 4 T-cell (CD4 T-cell) count, HIV is associated with a wide range of infections. Historically, PCP has been observed in HIV patients with AIDS or in those receiving immunosuppressive therapy. However, it should remain a differential diagnosis in patients who present with comorbidities and symptoms of acute lung disease.

## Introduction

Nicknamed an “AIDS-defining illness” for its association as an opportunistic lung infection caused by the fungus *Pneumocystis jirovecii* (formerly *P. carinii*), pneumocystis pneumonia (PCP) is a potentially lethal condition that typically occurs in HIV patients with a CD4 count < 200 cells/μL (reference range > 500 cells/mm³) [1]. It remains one of the leading causes of death in AIDS patients and is often treated prophylactically with trimethoprim/sulfamethoxazole (TMP/SMX) once the CD4 count drops below 200 cells/μL [2]. Additional conditions associated with increased susceptibility to this opportunistic pathogen include primary immunodeficiencies, malignancy-induced immunodeficiencies, chronic immunosuppressive therapy, and organ transplantation [3]. Although the precise pathogenesis is not fully understood, studies have shown that up to 90% of patients with non-HIV-related PCP had a history of glucocorticoid use [4]. Therefore, PCP should be suspected in any immunocompromised patient presenting with fever, non-productive cough, hypoxia, or worsening dyspnea [2]. The clinical presentation of PCP varies widely, from asymptomatic carriers to patients experiencing respiratory failure, depending on etiology, degree of immunosuppression, and presence of comorbidities [2]. Findings supportive of a PCP diagnosis include diffuse bilateral infiltrates on chest X-ray (CXR), patchy or nodular ground-glass opacities on CT, elevated lactate dehydrogenase (LDH), a positive β-D-glucan assay (a carbohydrate component in the fungal cell wall), hypoxia, and an elevated alveolar-arterial (A-a) oxygen gradient on pulmonary function testing [2]. The A-a gradient represents the difference between the partial pressure of oxygen in the alveoli (A) and that in arterial blood (a). The diagnosis of PCP is confirmed by detecting P. jirovecii in a sputum sample, via polymerase chain reaction (PCR) from bronchoalveolar lavage (BAL), or from lung tissue biopsy [5]. Treatment typically includes high-dose TMP/SMX for 21 days, with the addition of corticosteroids if the patient has hypoxia or an elevated A-a gradient [6]. We present a unique case of a 39-year-old male with HIV and an absolute CD4 count of 437 cells/μL (not on immunosuppressants), who presented with PCP.

## Case presentation

**Figure 1:**
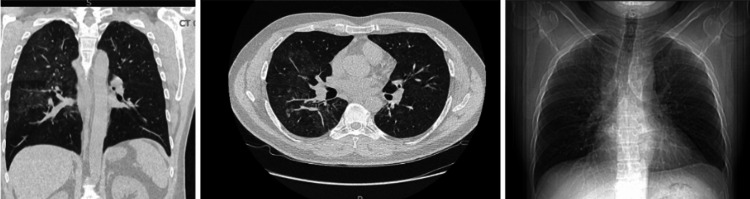
High-resolution CT and chest X-ray demonstrate ground-glass opacities and diffuse interstitial infiltrates in a patient with PCP. Courtesy of Leonard J. Chabert Medical Center. PCP: *Pneumocystis jirovecii* pneumonia.

A 39-year-old male with a past medical history (PMH) of HIV and chronic obstructive pulmonary disease presented to the emergency department with fever, cough producing clear or occasionally yellow sputum, nasal congestion, and shortness of breath that had started three days prior to presentation. The patient’s most recent CD4 count in 2019 was 947 cells/μL. He reported noncompliance with his antiretroviral therapy (ART) since 2019 and had not followed up with an infectious disease specialist. A blood culture was obtained and showed no growth. Chest X-ray (CXR) revealed bilateral patchy infiltrates (Figure 1). Due to diagnostic uncertainty, a chest CT scan was performed, revealing bilateral pulmonary consolidations consistent with multifocal pneumonia (Figure 1). The patient was isolated due to concern for potential tuberculosis (TB), which was ruled out by a negative QuantiFERON-TB Gold test. His respiratory infection panel was negative, and he was empirically started on ceftriaxone and azithromycin for presumed bacterial pneumonia. His CD4 count was found to be 437 cells/μL, and his HIV viral load was 47,950 copies/mL (reference range: <20-50 copies/mL). Respiratory cultures grew group B Streptococcus, and a Fungitell assay for β-D-glucan was positive at >500 pg/mL (reference range: <80 pg/mL). Despite receiving antibiotics, the patient’s high fevers persisted for seventy-two hours. A bronchoscopy with BAL was performed, and a PCR analysis of the sputum tested positive for *Pneumocystis jirovecii*. Additional testing revealed an alveolar-arterial (A-a) gradient of 52 mm Hg (reference range: 8-20 mm Hg). The patient was started on trimethoprim/sulfamethoxazole (TMP/SMX) on day seven of hospitalization. By day ten, his shortness of breath, cough, and fever had significantly improved. On hospital day twelve, he remained afebrile for over 48 hours and was discharged on oral TMP/SMX for 14 days, a short course of tapering prednisone, and ART. At a one-month follow-up, the patient denied any symptoms. A repeat CXR showed mild interstitial infiltrates, his CD4 count had improved to 611 cells/μL, his HIV viral load had decreased to 18,340 copies/mL, and the Fungitell β-D-glucan assay was negative.

## Discussion

A patient presenting with signs and symptoms of pneumonia, a PMH of chronic lung disease, and a condition leading to impaired immune function (HIV) is typically treated empirically with a combination of antibiotics (ceftriaxone and azithromycin) while awaiting results from sputum samples sent for testing [7]. This patient lacked follow-up with an infectious disease specialist and was noncompliant with HIV treatment. The association between medication nonadherence and the development of AIDS or opportunistic infections in patients with HIV is well established [8]. In addition, the patient did not appear to have other well-established risk factors for PCP (such as glucocorticoid use). Given this patient’s presentation and PMH, further investigation into his current viral load and CD4 T-cell count was warranted, as different opportunistic pathogens may cause various disease states depending on HIV viral load and CD4 count levels [9]. Necessary isolation precautions were implemented for a potential TB infection, as HIV may alter the typical disease patterns of TB [10]. The CXR was inconclusive, and a high-resolution CT scan of the chest was performed as indicated [11]. As mentioned in the introduction, radiographic evidence of ground-glass opacities and diffuse interstitial infiltrates is a classic finding in patients with PCP [2]. In patients with suspected pneumonia, a lack of clinical improvement or response to empiric antibiotic therapy warrants pulmonology consultation for BAL and further investigation [12]. To our surprise, the PCR analysis of the sputum obtained via BAL was positive for *P. jirovecii*. Given the patient’s elevated A-a gradient, he was started on TMP/SMX, oxygen therapy, and intravenous prednisolone. He showed significant symptomatic improvement after three days of treatment. He was discharged after remaining afebrile for at least 48 hours, with a 14-day course of oral TMP/SMX and a tapering dose of oral prednisolone [13]. The observed clinical, radiographic, and laboratory improvement during follow-up further supported the diagnosis of PCP. Thus, the care team concluded that this was a unique case of PCP.

## Conclusions

We report a case of pneumocystis pneumonia in an HIV patient with a CD4 count of 437 cells/μL who was not on immunosuppressive therapy, highlighting a rare but increasingly recognized presentation of this condition.
